# Big data- and artificial intelligence-based hot-spot analysis of COVID-19: Gauteng, South Africa, as a case study

**DOI:** 10.1186/s12911-023-02098-3

**Published:** 2023-01-26

**Authors:** Benjamin Lieberman, Jude Dzevela Kong, Roy Gusinow, Ali Asgary, Nicola Luigi Bragazzi, Joshua Choma, Salah-Eddine Dahbi, Kentaro Hayashi, Deepak Kar, Mary Kawonga, Mduduzi Mbada, Kgomotso Monnakgotla, James Orbinski, Xifeng Ruan, Finn Stevenson, Jianhong Wu, Bruce Mellado

**Affiliations:** 1 School of Physics and Institute for Collider Particle Physics, University of the Witwatersrand, Johannesburg, South Africa; 2 Disaster and Emergency Management, School of Administrative Studies and Advanced Disaster, Emergency and Rapid-response Simulation, York University, Toronto, Canada; 3 Department of Mathematics and Statistics, York University, Toronto, Canada; 4 Laboratory for Industrial and Applied Mathematics (LIAM), York University, Toronto, Canada; 5 School of Computer Science and Applied Mathematics, University of the Witwatersrand, Johannesburg, South Africa; 6 School of Public Health, University of the Witwatersrand, Johannesburg, South Africa; 7 Gauteng Provincial Department of Health, Johannesburg, South Africa; 8 Africa-Canada Artificial Intelligence and Data Innovation Consortium (ACADIC), Toronto, Canada; 9 Gauteng Office of the Premier, Johannesburg, South Africa; 10 iThemba LABS, National Research Foundation, Somerset West, South Africa; 11 Dahdaleh Institute for Global Health Research, York University, Toronto, Canada

**Keywords:** COVID-19, South Africa, Gauteng department of health, Hot-spot, Risk adjusted strategy, Control intervention, Big data, Artificial intelligence

## Abstract

The coronavirus disease 2019 (COVID-19) has developed into a pandemic. Data-driven techniques can be used to inform and guide public health decision- and policy-makers. In generalizing the spread of a virus over a large area, such as a province, it must be assumed that the transmission occurs as a stochastic process. It is therefore very difficult for policy and decision makers to understand and visualize the location specific dynamics of the virus on a more granular level. A primary concern is exposing local virus hot-spots, in order to inform and implement non-pharmaceutical interventions. A hot-spot is defined as an area experiencing exponential growth relative to the generalised growth of the pandemic. This paper uses the first and second waves of the COVID-19 epidemic in Gauteng Province, South Africa, as a case study. The study aims provide a data-driven methodology and comprehensive case study to expose location specific virus dynamics within a given area. The methodology uses an unsupervised Gaussian Mixture model to cluster cases at a desired granularity. This is combined with an epidemiological analysis to quantify each cluster’s severity, progression and whether it can be defined as a hot-spot.

## Background

In late December 2019, a novel coronavirus, named “Severe Acute Respiratory Syndrome-related Coronavirus type 2” (SARS-CoV-2), emerged in the city of Wuhan, Hubei province of People’s Republic of China [1]. The virus rapidly spread by the 11th of March 2020, resulting in a confirmed global pandemic, known as “Coronavirus Disease 2019” (COVID-19). As of the 5th of March 2021, the virus was affecting more than 218 countries, with the total number of confirmed cases exceeding 116 million and approximately 2.6 million fatalities worldwide being attributed to the effects of the virus. A large, worldwide modelling effort is currently underway to improve public health policy decision-making with regards to the still ongoing COVID-19 pandemic [2]. Many research groups and national response teams have looked into country specific intervention strategies and the effects they have on the transmission rate of the virus as well as the impact of pre-existing country characteristics on the transmission rate [3, 4].

On the 5th of March 2020, South Africa recorded its first COVID-19 case and three weeks later, on the 27th of March, South Africa entered a full government-enforced lockdown [5]. This formed part of a five-tier risk-adjusted alert levels system [6]. The full list of South Africa’s moves between lockdown levels can be seen in Table 1, [7]. The first wave of COVID-19 continued in South Africa until October 2020 where the number of new cases had settled to a manageable amount. By late November 2020, South Africa’s number of cases started to increase, and the second wave of the pandemic began. The risk-adjusted system implemented allowed a controlled reopening/closing of the economy influenced by a set of factors, including the virus transmission rate, number of infectious cases, capacity of health facilities, the extent of the effectiveness of the implemented public health interventions and the economic and societal impact of continued restrictions.

**Table 1:** South Africa’s alert level progression for waves 1,2 and 3

Alert level	Wave	Start date	Total cases	Recoveries	Fatalities
5	1	27 March 2020	927	12	0
4	1	1 May 2020	5951	2382	116
3	1	1 June 2020	34,357	17,291	705
2	1	18 August 2020	592,144	485,468	12264
1	2	21 September 2020	661,936	591,208	15,992
3	2	29 December 2020	1,021,451	858,456	27,568
1	2	1 March 2021	1,513,959	1,431,336	50,077
2	3	31 May 2021	1,665,617	1,559,337	56,506
3	3	16 June 2021	1,774,312	1,620,317	58,223

The University of Witwatersrand and iThemba LABS COVID-19 modelling group have formed part of the Gauteng Premier’s COVID-19 Advisory Committee, providing an in-depth analysis of the province’s progress in the pandemic [8]. As part of the Gauteng Premier’s COVID-19 Advisory Committee, our modeling efforts provide information that government stakeholders use to inform their decisions, thus allowing a statistical ground for changes in alert levels and distribution of resources.

COVID-19 data contain many complexities that must be taken into account when extracting information to guide public health decision- and policy-makers [9]. This complexity includes factors such as the large number of misclassified or under-reported infections, inconsistency and limitations in testing as well as fluctuating infection and fatality rates as influenced by social/behavioral dynamics.

As this data is the basis for modeling and therefore, informing decisions around the risk-adjusted policies, understanding and accommodating these complexities in the model is vital. In generalising the spread of a virus over a large area, such as a province, it must be assumed that the transmission occurs as a stochastic process. This statistically random spread of a virus through a population is the core of the majority of Susceptible-Infectious-Recovered-Deceased (SIRD) models and is dependent on factors such as number of infected cases, infection rate, level of social interactions, susceptible population and total population [8]. However, the spread of COVID-19 and therefore, the data representing the virus progression do not always conform to a stochastic model. In this paper, we will focus on the most influential non-stochastic dynamics of COVID-19, hot-spots.

A virus hot-spot can be defined as a cluster of cases within an area whose spreading dynamics do not conform to the general growth of the pandemic, exhibiting an exponential, short-lived growth. As the collections of cases clustered as hot-spots described in this paper do not conform to the macro-dynamics of their location, they need to be clearly defined and understood in order to accurately understand and model the virus progression. The geo-localization and clustering analyses of cases for this purpose are therefore, vital and can be done using advanced artificial intelligence (AI) geo-clustering methods. This clustering approach can be used to define individual clusters as hot-spots and allows the corresponding cases to be removed from the stochastic model - providing stochastic predictions that are not biased by the hot-spot dynamics [7].

The structure of this paper is as follows, firstly the data and data collection is described followed by the methodology of the clustering algorithm used. The paper continues to investigate the results of the clustering together with the methodologies and parameterisation of the clusters. The parameterisation of the clusters includes whether or not a cluster is a hot-spot, the temporal progression and the severity of the cluster. Finally the applications of the classification and parameterisation are evaluated and validated using the second wave.

In the existing scholarly literature, some studies have performed a hot-spot analysis of COVID-19. For instance, Shariati and colleagues [10] have computed Anselin Local Moran’s I indices to identify high- and low-risk clusters of COVID-19 worldwide. Authors were able to locate San Marino and Italy as territories characterized by a dramatically high toll of deaths, with infectious hot-spots widespread in Northern Africa as well as Southern, Northern and Western Europe. Noteworthy, infectious cases occurring in these hot-spots represent about 70 percent of all global infectious cases.

Other hot-spot analyses have been carried out at the nation level. Mo and coworkers [11] coupled local outlier analysis with hot-spot analysis based on space-time cube metrics in mainland China. Authors were able to demonstrate a rather quick, uneven spreading of the outbreak from the cities of Wuhan and Shiyan to the neighbouring areas and provinces.

In Italy, combining a variety of geospatial analytical methods (spatial auto-correlation, spatio-temporal clustering and kernel density techniques), infodemiology (Google Trends and web searches analysis) and AI methods (machine learning and Adaboost algorithm for single?factor modelling), Niu and collaborators [12] were able to provide an in-depth assessment of the COVID-19 outbreak, in terms of its distribution and spreading characteristics. Hot-spots could be identified mainly in northern Italy.

Purwanto and colleagues [13] explored COVID-19 distribution patterns in East Java (Indonesia). Authors were able to identify Surabaya as major hot-spot, from which the outbreak reached cities characterized by high density of roads, food venues, and commercial and financial facilities.

AI models in healthcare are not limited to epidemics and are utilised for various applications including drug-drug interactions [14] and the identification of salient sites in epigenetics [15].

In the present investigation, we have provided a robust statistical method for distinguishing between hot-spots and areas characterized by stochastic spreading of COVID-19 cases. We applied this analytical framework to the first and second waves, taking Gauteng province, South Africa, as a case study. These methods are general-purpose and can be, as such, applied to other countries as well.

The primary aim of this paper is for policy makers and local population to visualise and understand the location specific dynamics of the virus. This is vitally important for implementation of non-pharmaceutical intervention on a local level.

## Materials and methods

In order to expose the location specific COVID-19 dynamics within a given area, the following methodology is used. Firstly the geo-coded case data is processed, for the area of study. The data is clustered using Gaussian Mixture Models, grouping cases by their locations, at a selected granularity. Once the cases are clustered, the parameters of logistic growth are calculated for each cluster to reflect the area specific virus progression. An analysis of the cluster dynamics can then be used to calibrate/define criterion for clusters to be considered hot-spots, the extent to which the cluster is active and a measurement of risk associated with it. During the first wave of the pandemic, the definitions must be redefined and improved as new data is made available. However after the completion of the first wave the first wave data can be used to produce criterion reflective of the area of analysis and can therefore be used in the analysis of subsequent waves. In this paper it is assumed that the initial wave of the pandemic is complete and is utilised in the calibration of cluster definitions.

### Study area

**Fig. 1:**
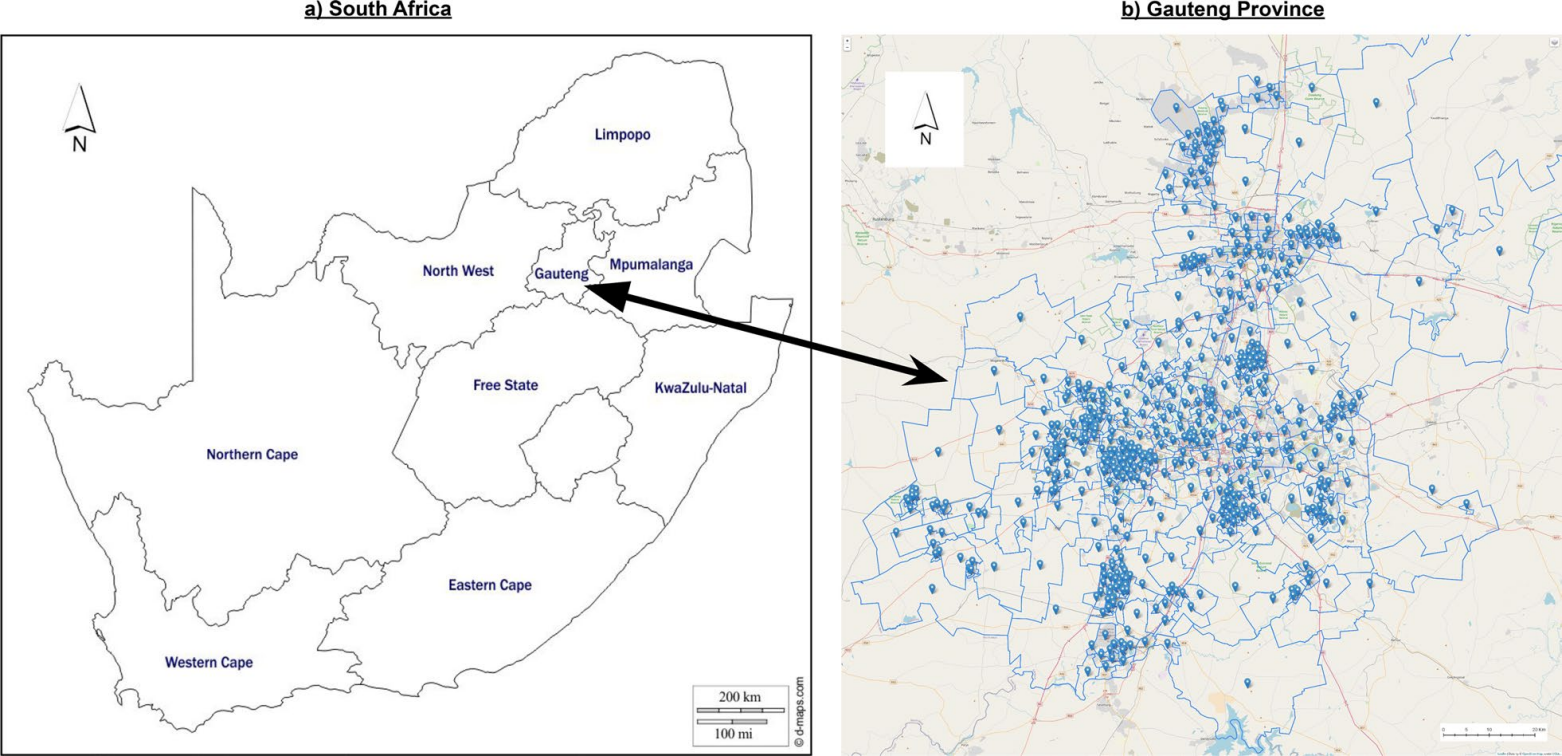
Study area. **a** Regional map of South Africa. **b** Map of Gauteng province showing breakdown of wards

Gauteng ($26.2708^{\circ }$ S, $28.1123^{\circ }$ E) is one of the nine provinces of South Africa, shown in Fig. 1. Although Gauteng is the smallest of South Africa’s provinces, with an area of 18,176 km$^{2}$, it is home to approximateley 16 million people, more than a quarter of the countries population. South Africa’s largest city, Johannesburg, as well as it’s administrative capital, Tshwane, are situated in Gauteng.

### Data processing

The data required for the hot-spot geo-localization analysis needs to be of a high level of detail. Therefore, for this study anonymized data provided by the Gauteng Department of Health is used. The data contains features including; Case ID, recorded address, test date and geo-localization data (including latitude and longitude coordinates). The National Institute of Communicable diseases (NICD) collates the daily data of SARS-COV-2 tests that are conducted both in public and private laboratories across South Africa. The NICD publishes a daily report detailing the provincial breakdown of the COVID-19 Cases in South Africa [16].

The NICD’s daily data is fed into the Gauteng Department of Health’s data management system where each case is geo-coded to their specific geo-location before being de-identified (for anonymity of cases). The processed data is then sent to external organisations, such as ourselves, for analysis. Before the data can be used for clustering, a final filtering is done to remove any geo-localization data that has an incorrect address recorded or an issue interpreting/processing the address.

For the first wave in Gauteng, March–October 2020, 218720 geo-coded case samples were used. During the second wave progression, November 2020 to February 2021, 191750 samples were used. As of May 2021, the geo-coded case data was no longer made available.

### Clustering cases by geo-location

In order to analyse the area distribution of COVID-19 cases, AI techniques provide an excellent tool in grouping cases geographically. In this paper we focus on the unsupervised machine learning method, Gaussian mixture models. This model allows us to group cases based on their location. The output clusters can therefore be used for analyse and to model the dynamics of the virus within the determined area. The generation of the Gaussian Mixture Model distributions and corresponding HTML maps were implemented in Python 2.7, utilising the Sckit-learn API package [17].

#### AI and clustering: Gaussian mixture model (GMM)

**Fig. 2:**
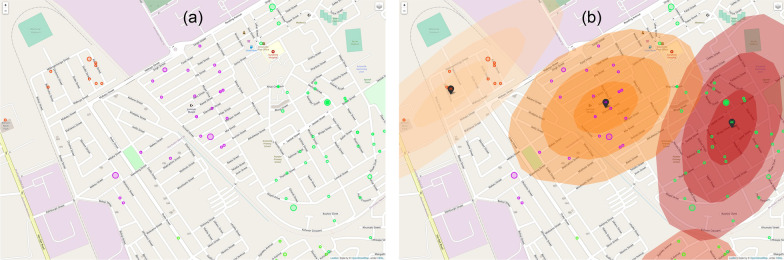
Map visualisation of Gaussian Mixture Model clustering of COVID-19 cases in Gauteng. **a** Map showing case data input for the model. **b** Map showing case data and clustering model output

The given problem is using the location of residence of each COVID-19 case in Gauteng to produce clusters. Once defined, these clusters can be analysed and accurately labelled as hot-spots or non-hot-spots. There are various clustering methods where unsupervised machine learning algorithms are implemented to solve a 2-dimensional (latitude/longitude co-ordinates) problem. After evaluating various methods including the k-means algorithm, the Gaussian mixture model was chosen. Gaussian Mixture models provide a probability-based approach to the likelihood of a COVID cases being within a cluster by producing a 2-dimensional Gaussian probability model overlaid onto the Gauteng map area, shown in Fig. 2. The clusters produced can overlap with each other, which encapsulates the possibility that hot-spots may very well also overlap with each other. The corresponding weight, $\phi$, generated for each cluster, provides an estimate of the importance of the cluster, as well as another variable for filtering false clusters from actual hot-spots [18].

A Gaussian Mixture model is an algorithm which operates by generating *k* 2-dimensional Gaussian probability distributions, where *k* is a specified hyper-parameter. Thus, we are required to generate means, $\mu _{j}$, covariance $\Sigma _j$ and weighting, $\phi _j$, where the index specifies the jth Gaussian cluster. So, the probability of a new case, *p*(*x*), occurring at a given point *x* is a linear combination of probabilities from all the generated clusters:

1 $$\begin{aligned} p(x) = \sum ^{k}_{j=1} \phi _j N(x|\mu _j,\Sigma _j), \end{aligned}$$

where *N* is the normal distribution. We generate the set of normal distributions (with associated weights, means and covariances) with an algorithm which optimally fits the probability distributions given the set of already known COVID-19 cases and their coordinates. In order to generate *k*-Gaussian probability distributions, the Expectation-Maximisation algorithm is employed. At the expectation step, we calculate the probability that a point $x_i$ is generated by the jth Gaussian for all *k* distributions:

2 $$\begin{aligned} \gamma _{ij} = \frac{\phi _j N(x_i|\mu _j, \Sigma _j)}{\sum ^{k}_{q=1}\phi _j N(x_i|\mu _1,\Sigma _q)} \end{aligned}$$

In the maximisation step, the probabilities $\gamma _{ij}$ are used to generate new cluster parameters. That is, new mean $\mu _j$, covariance $\Sigma _j$ and weight $\phi _j$ are updated as follows:

3 $$\begin{aligned} \phi _j = \sum ^{N}_{i}\frac{\gamma _{ij}}{N}, \, \mu _j = \sum ^{N}_{i}\frac{\sum ^{N}_{i}\gamma _{ij}x_{i}}{\sum ^{N}_{i}\gamma _{ij}}, \, \Sigma ^{2}_{j} = \frac{\sum ^{N}_{i}\gamma _{ij}(x_i - \mu _j)(x_i - \mu _j)^T}{\sum ^{N}_{i}\gamma _{ij}} \end{aligned}$$

These steps are iterated through until the convergence criteria are met. In our case, the variable $x = \{x,y\}$ is the set of longitudinal, *y* and latitudinal coordinate, *x*.

**Fig. 3:**
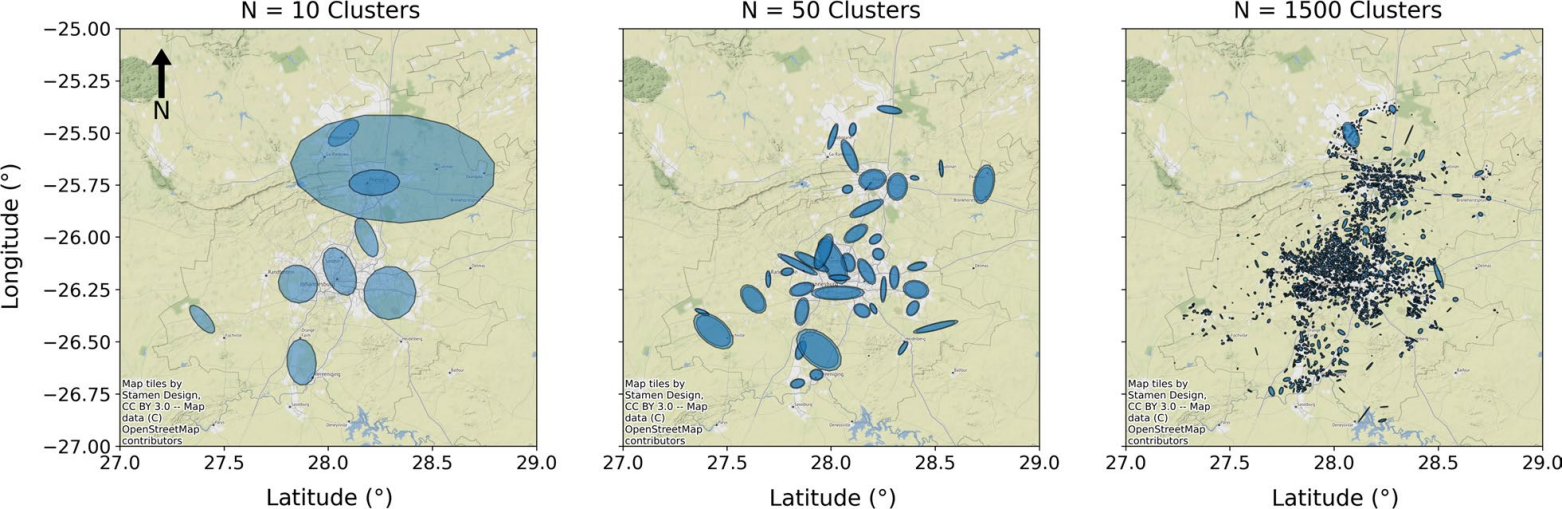
Visualisation of GMM cluster output for different number of clusters. **a** 10 Clusters. **b** 50 Clusters. **c** 1500 Clusters

When applying the GMM algorithm for the clustering of cases in Gauteng, the number of clusters generated is an important metric. The number of clusters must be selected to best describe the specific virus dynamics, as visualised in Fig. 3. In the case of Gauteng Province, it was advantageous to have a cluster size approximately the size of a suburb, with at least an average of 100 cases per cluster. It was determined that 1500 clusters was the optimal number of clusters for the Gauteng province. This led to the average cluster area being 1.9 km$^{2}$ and an average of 146 cases/cluster for the period of the first wave. Therefore providing the highest level detail possible while maintaining sufficient statistics.

#### Susceptible-infectious curve

Once the latent variables of the Gaussian probabilities distributions (weights, means, standard deviation) have been found through the processing of COVID-19 cases in Gauteng, it is important to verify which clusters are hot-spots, or highly infectious areas/districts of the province. In order to accomplish this, the time dependent progression of cases is inspected for each cluster independently. That is, the cumulative number of cases was computed as a function of the date the patients were first recorded to have contracted the virus.

**Fig. 4:**
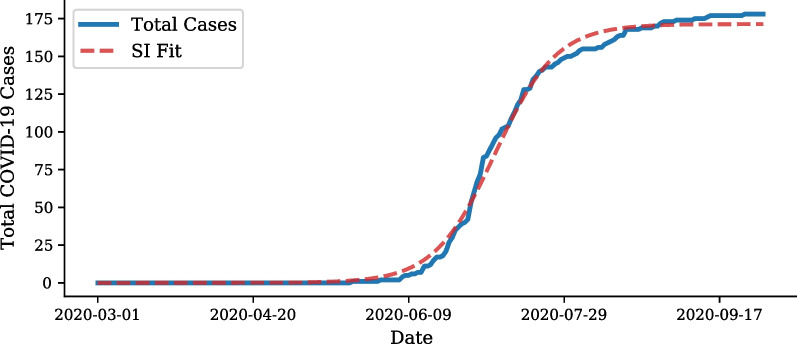
SI curve example. Example of SI curve fit to cumulative COVID-19 cases in a cluster

An aspect to consider is whether the clusters found follow the Susceptible-Infection (SI) Curve, which model the number of susceptible people who get infected, SI(t), over time, *t*, within a given area/cluster. The SI equation is as follows:

4 $$\begin{aligned} SI(t) = \frac{SI_0}{1 + e^{SI_1(t-SI_2)}} \end{aligned}$$

where $SI_0$ is the total number of predicted cases within a cluster once it has saturated the susceptible population, $SI_1$ represents the rate of infection of the virus, and $SI_2$ is the number of days before the peak of growth of the cluster. An example of the SI Curve, fit to cumulative cases of a single cluster, is shown in Fig. 4. This function is a solution to the logistic differential equation, a simple system which describes the number of infected cases in a given population. The model is applicable as we expect a small increase of infection cases in the early stages of a susceptible population, and then a sharp increase as the disease spreads rapidly throughout the cluster. A plateau is expected once all susceptible people within a cluster are infected. The SI curve can therefore, be fitted to the time-series of each cluster in order to generate the cluster’s localised virus parameters. A poorly fit SI curve can indicate that the cluster is not a COVID-19 hot-spot, as it does not follow an accurate description of disease spread.

Once the cases throughout Gauteng province have been clustered and described, each cluster can be described through the following parameters; Total Cumulative Cases ($N_{TC}$), 1st and 2nd standard deviation area ($A_{1sd}$ and $A_{2sd}$, respectively), the susceptible-infection parameters ($SI_0$, $SI_1$ and $SI_2$) and the ward and municipality where the cluster is located.

### Cluster analysis and hot-spot definition using the first wave

In order to understand the cluster level COVID-19 dynamics in Gauteng, the GMM clustering method, described in the previous section, is applied to the Gauteng case data for the calibration period available. The SI parameters are then calculated, using Eq. 4, for the temporal case progression of each cluster. Using the cluster parameters, of available calibration data, the following criterion are designed to analyse and categorise hot-spot clusters. The following definition uses the entirety of the first wave to define the criterion which can thereafter be applied to proceeding waves occurring in Gauteng. The first wave data used for calibration contains 218720 samples, leading to an average of 146 cases per cluster.

#### Hot-spot classification on density

**Fig. 5:**
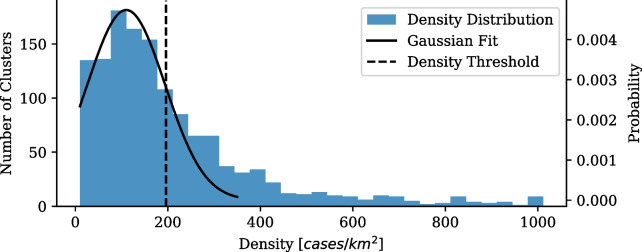
Gauteng first wave cluster density distribution. Density distribution for COVID-19 clusters for the period of the first wave in Gauteng

The density distribution of the first wave clusters, shown in Fig. 5, forms a Gaussian like shape at low densities, 0–350 cases/km$^2$, and a sporadic tail of high densities, 350 to more than 30000 cases/km$^2$. The uniformity of low-density clusters is found to be associated with expected growth. When cutting the densities at the one Sigma interval we are able to produce a density threshold, $\rho _{th}$, of 196.05 cases/km$^2$. Clusters with densities greater than the threshold are found to have rapid, non-stochastic growth. This density threshold, therefore, allows us to define hot-spot clusters as any cluster whose density exceeds the determined density threshold.

#### Hot-spot cluster activity definition

**Fig. 6:**
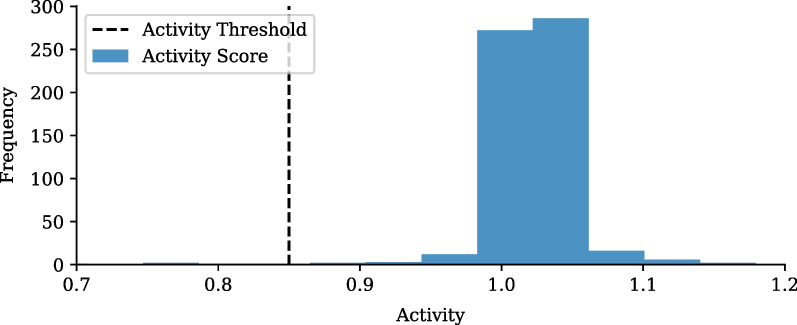
Gauteng first wave cluster activity distribution. Activity distribution for COVID-19 clusters after the completion of the first wave in Gauteng

Once a hot-spot cluster’s total cases reach the plateau or pass the peak of a surge, it can be said that the dynamics of the cluster are no longer that of a hot-spot. The activity of a cluster at any point in time can, therefore, be quantified as the ratio of the total cases in the cluster, at the respective time, divided by the cluster’s total predicted cases, $SI_0$, described in Criteria 5:

5 $$\begin{aligned} \frac{N_{TC}(t)}{SI_0} < L_{th}, \end{aligned}$$

where the activity threshold, $L_{th}$, represents the upper bound on actively growing clusters. The activity threshold assumes that only $1\%$ of clusters remain active in the period after the first wave as almost all clusters have returned to normal dynamics. Therefore, as shown in the activity distribution, Fig. 6, the activity threshold for Gauteng based on the first wave, is determined to be 0.85.

#### Risk index definition

**Fig. 7:**
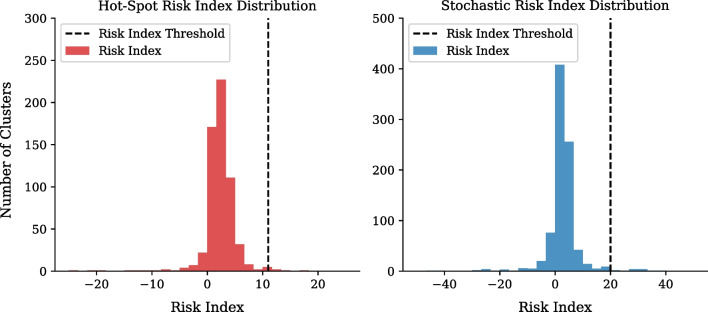
Gauteng first wave risk index distributions. Risk Index distributions broken-down into hot-spot and stochastic clusters

The risk index, RI, quantifies the deviation of the data from the hypothesis of a single wave. It therefore gives a measure of the risk of a cluster behaving non-stochastically for a future wave. The risk index is defined in Criterion 6 and 7.

6 $$\begin{aligned} A(t)= & {} 100\cdot \left( \frac{N_{TC}(t)-SI_0(t)}{SI_0(t)}\right) , \,\, B(t) = 10 \cdot \left( 1 + \frac{SI_0(t)}{N_{TC}(t)}\right) ^{-1}, \end{aligned}$$

7 $$\begin{aligned} RI= & {} \left\{ \begin{array}{@{}ll@{}} A(t) + B(t), &{} \text {if}\,\ B(t) > 8, \\ A(t), &{} \text {if}\,\ B(t) \le 8. \end{array}\right. \end{aligned}$$

where $N_{TC}(t)$ and $SI_0(t)$ are the total cases, and total predicted cases in the cluster at a given time, t, respectively. Applying Criterion 7 to both the hot-spot and stochastic clusters independently, leads to the distribution shown in Fig. 7. The *RI* threshold assumes that only $1\%$ of clusters are high risk in the subsequent period of the first wave with a corresponding proportional error. Figure 7 therefore shows the risk index at which a cluster can be defined as high risk location within Gauteng Province.

Therefore, in the analysis of future waves in Gauteng, a hot-spot cluster with a *RI* greater than 11 can be classified as a high risk hot-spot. Similarly a non-hot-spot cluster with a RI greater than 20 can be classified as a developing high risk cluster.

### Applying calibrated cluster definitions to subsequent data

The criterion for clusters to be labeled as a hot-spot, as well as the activity and risk index definition are calibrated using historic data. In this paper the first wave data is used for calibration. From the start of a subsequent wave, cases are clustered as they are made available and are analysed using the calibrated criterion. For each iteration of new data available, the clustering and analysis are re-applied. Therefore as new data becomes available, all of the samples, for the period of analysis, are used for clustering independently to previously determined clusters. For each point in time during the progression of subsequent waves, the study is able to expose the location, temporal progression and severity of active hot-spots as well as cluster’s with high likelihood of developing into hot-spots.

## Results and discussion

### Analysis of cluster definition calibration on Gauteng Province’s first wave

We calibrated the density criterion to the first wave of COVID-19 cases in Gauteng Province where $\rho _{cluster}(t)$ is the case density of a given cluster on a given day and $\rho _{th}$ is the minimum density stipulating hot-spot dynamics. Out of 1500 clusters, once split on the density threshold 607 of the clusters are defined as hot-spots and the remaining 893 clusters are defined as normal clusters.

**Fig. 8:**
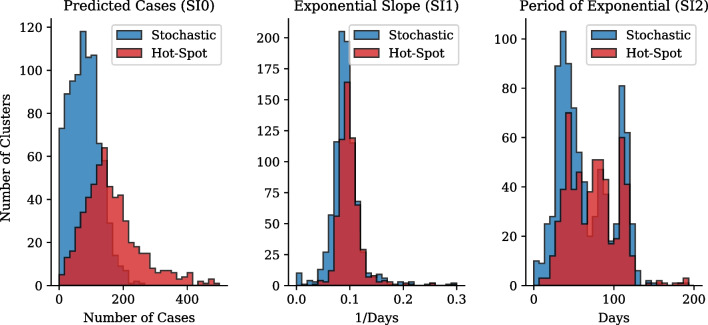
Gauteng first wave cluster parameter distribution Comparison. Susceptible-Infectious parameter distributions for clusters

In order to evaluate this definition further we compare the susceptible-infection parameters of the clusters defined as hot-spots against the stochastic or non-hot-spot clusters. Figure 8 shows that hot-spot clusters have on average an increased number of total cases, $\pm \,180$, compared to the stochastic clusters, $\pm \,90$. Hot-Spot clusters also have a slightly increased exponential slope with a period of $\pm \,10$ days where stochastic clusters period of exponential slope can be seen to be $\pm \,11$ days.

**Fig. 9:**
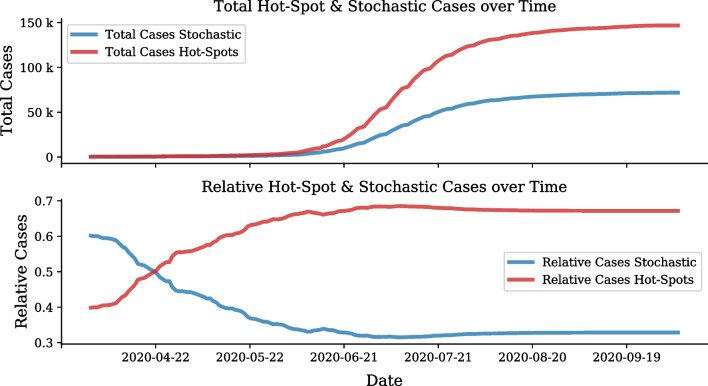
Number of hot-spot cases over time during the first wave. Comparison of hot-spot and stochastic growth using number of cases per day

An evaluation of this hot-spot definition can be done using a comparison of the total cases in stochastic clusters and hot-spot clusters during the first wave. Figure 9 reflects that during the first wave approximately two thirds of the cases in Gauteng occurred in hot-spot clusters.

**Fig. 10:**
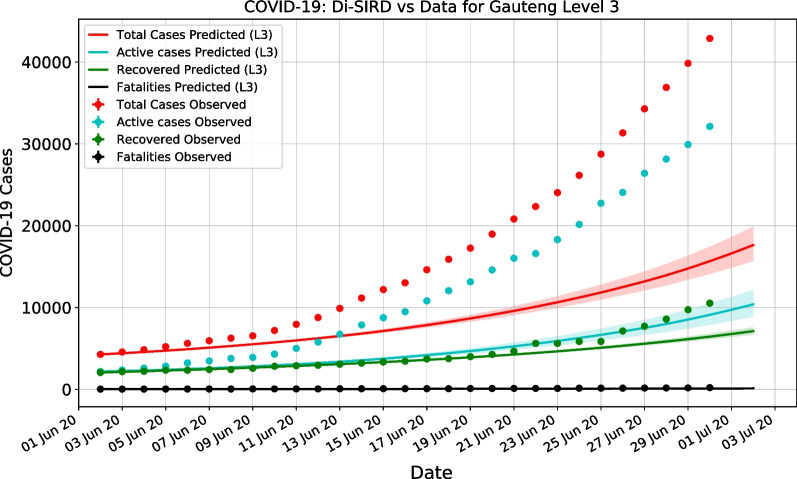
Example of first wave stochastic prediction versus data. Di-SIRD model stochastic prediction vs data for Gauteng for June 2020

This case distribution shows excellent coherence with first wave predictions (Using a Di-SIRD linear control model [7]) compared to data, as shown in Fig. 10. This example of a stochastic prediction demonstrates how the emergence of hot-spots in June 2020 did not follow the expected stochastic progression of the virus.

Therefore, it can be seen that the density cut-off value of 196 cases/km$^2$, defining hot-spot clusters, successfully is able to extract the clusters growing more exponentially and sporadically from those with a more uniform, random growth.

#### Hot-spot activity analysis

**Fig. 11:**
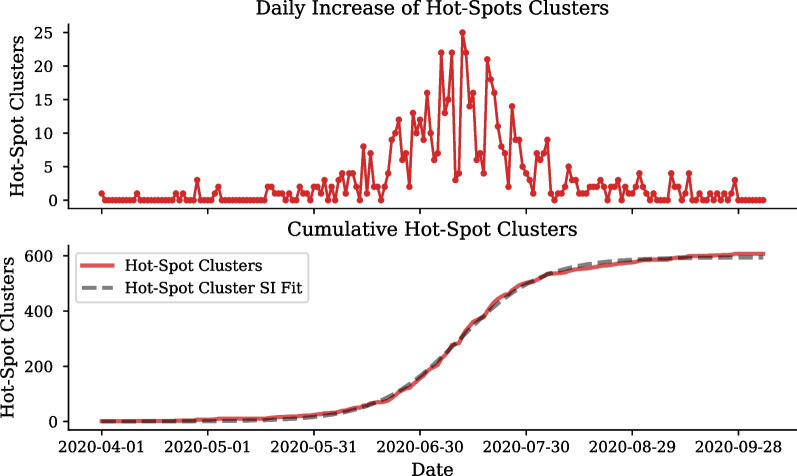
First wave cumulative and emerging COVID-19 hot-spot clusters in Gauteng. Number of new clusters developing into hot-spots (top). Total number of clusters defined as hot-spots for the first wave in Gauteng (bottom)

**Fig. 12:**
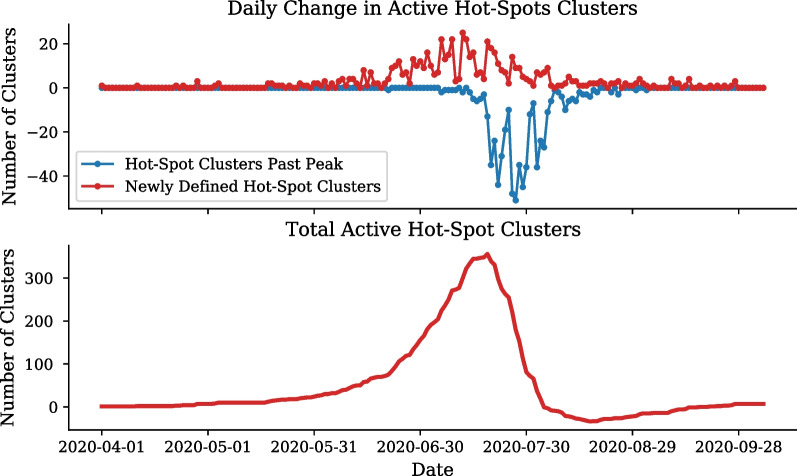
Daily number of active hot-spot clusters. Number of clusters developing into active hot-spots and number of hot-spot clusters becoming no longer active (top). Total number of clusters developing into active hot-spots (bottom)

The time dependent evolution of newly defined hot-spots as well as hot-spots that are returning to stochastic dynamics, during the first wave, can be analysed using Criterion 5. These dynamics are visualised in Figs. 11 and 12, respectively.

To understand the growth of the hot-spot clusters an SI curve is fit to the cumulative number of hot-spot clusters shown in Fig. 11. The daily increase of hot-spot clusters peaks in mid-July, which is confirmed by the $SI_2$ parameter which determines the inflection of exponential growth to occur on the 10th of July, 101 days after the 1st of April. The cumulative hot-spot clusters reaches its plateau in mid-August coinciding with South Africa’s move from level 3 to level 2, with 594 of the total 1, 500 clusters having already developed into hot-spots. The SI fit to the cumulative number of hot-spot clusters describes the period of the exponential growth to be approximately 12 days ($\frac{1}{SI_1}$).

Figure 12 shows not only the emergence of hot-spot clusters but also when hot-spots progress back to a stochastic dynamics, described by Criterion 5. From mid-July, the majority of hot-spot clusters begin to reach their peak progression, and therefore, progress back to stochastic clusters. By the end of August a maximum of 39 hot-spots have reached their peak and by the end of September all but 21 cluster have progressed back to normal dynamics.

### Implementation of hot-spot definition on Gauteng Province’s second wave

**Fig. 13:**
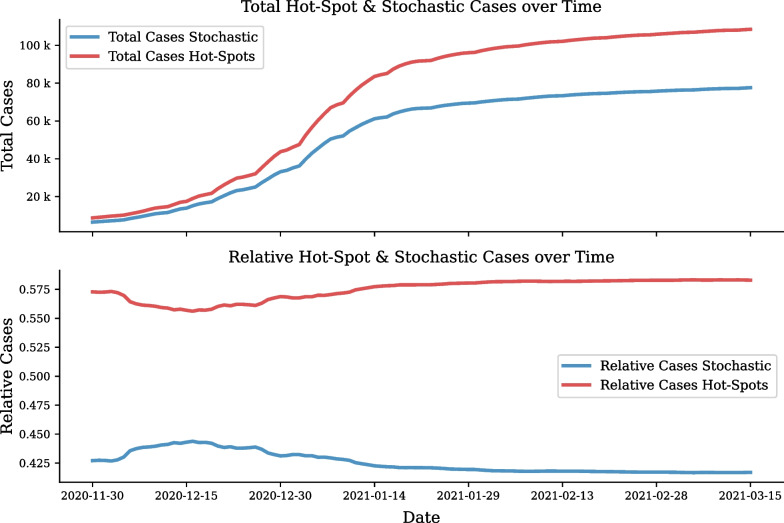
Number of hot-spot cases over time during the second wave. Comparison of hot-spot and stochastic growth using number of cases per day

Once the cluster definitions are calibrated on historical data, they can be applied to subsequent data. As the majority of hot-spots manifest during pandemic waves, it is ideal to implement the analysis on data specific to a wave of interest. In this section the first wave definitions are applied to the second wave. During the second wave period clustering and analysis was applied weekly on receiving data. During this period, a total of 191,750 cases were analysed. The case progression attributed to hot-spot and normal clusters is shown in Fig. 13. It can be seen that during the second wave nearly $60\%$ of cases occurred in hot-spots.

**Fig. 14:**
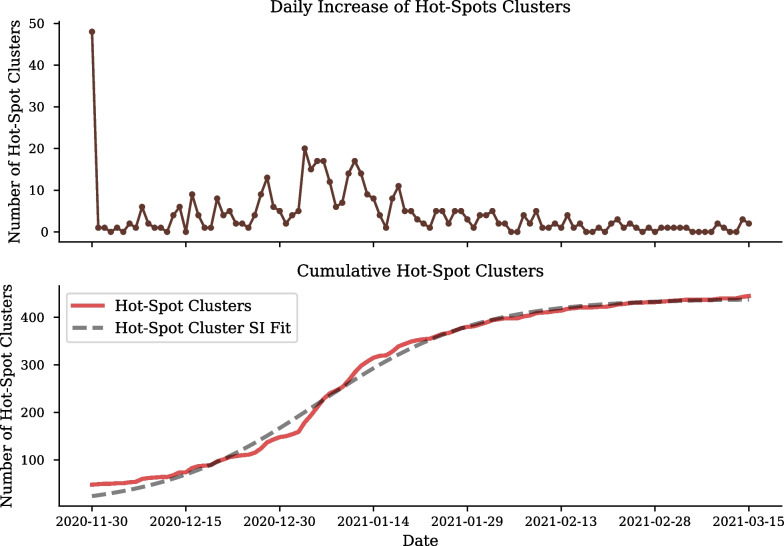
Second wave cumulative and emerging COVID-19 hot-spot clusters in Gauteng. Number of new clusters developing into hot-spots (top). Total number of clusters defined as hot-spots for the second wave in Gauteng (bottom)

A total of 461 clusters were categorised as hot-spot clusters and 1039 clusters had normal growth dynamics, as shown in Fig. 14.

At the start of the second wave, 11 November 2020, 48 clusters were found to be active hot-spots. At the end of the second wave, 15 March 2021, 7 hot-spots remained active and 454 clusters had returned to normal dynamics.

When looking at the clusters in terms of their risk index, 313 of the hot-spot clusters were classified as high-risk and 263 stochastic clusters were classified as emerging clusters. More than $90\%$ of clusters initially labeled emerging stochastic clusters, developed into hot-spot locations during the second wave. The risk index was used to inform local stake-holders of the wards and municipalities associated with the most severe hot-spot clusters. The most severe wards during the second wave were found to be ward 74804016 in Merafong City municipality, wards 79700088 and 79700005 in Ekurhuleni municipality, wards 74201033, 74201007, 74201008, 74201036, 74201010 and 74201024 in Emfuleni municipality, wards 79900105, 79900082 and 79900061 in City of Tshwane municipality and 79800053 and 79800061 in the City of Johannesburg municipality.

As all clusters activity, severity and location were expose at each interval of analysis during the second wave, both provincial and municipal stake-holders were able to visualise and sort cluster’s of interest to expose location specific virus dynamics.

### Exposure and applications of hot-spots

The definition and parameterization of clustered cases provides various applications in informing stakeholders in their decisions related to COVID-19 interventions and preventative measures. The following section discusses two of these applications. The first and most important role is to expose locations of extreme virus dynamics, in order to inform intervention strategies, advance social awareness and the adoption of proper behaviors. The second application allows for the hot-spot dynamics to be integrated into epidemiological models.

#### Exposing hot-spot and high risk clusters

The primary need for COVID-19 Hot-Spot classification is to target clusters/areas where non-conforming, exponential growth is occurring. Using the definition of hot-spot clusters developed in this paper, clusters can effectively be classified and their progression and dynamics described. Table 2 summarizes descriptive parameters of a classified cluster.

**Table 2:** Summary of specifications of classified clusters

Hot-spot classification	Cluster activity	Risk index
If cluster can be defined as a hot-spot or not	The time dependent progression of the cluster	The severity of infection rate and scale of clusters

**Fig. 15:**
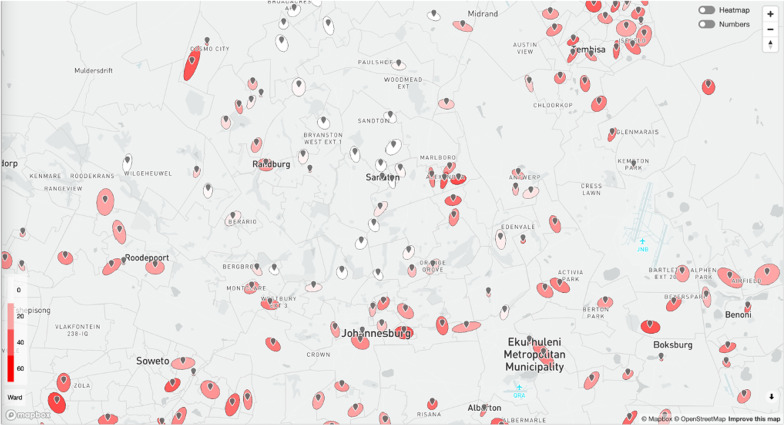
Hot-spot visualisation. Hot-Spot visualisation on gpcoronavirus.co.za. Courtesy of IBM South Africa

These three parameters describing each cluster are able to inform stakeholders not only on what areas are considered COVID-19 high growth areas but also the period of time the cluster will last and how severe the dynamics of the cluster is. This can then be visualised in an interactive map for stakeholders as shown in Fig. 15. The colour code of the clusters visually displays the severity using the *RI*.

Emerging spatio-temporal hot-spot analysis is of crucial importance for public health policy- and decision-makers and can provide valuable information that would not be possible to achieve with other techniques, enabling to capture specific clustering patterns in terms of particular districts and areas that would be otherwise classified as being at low risk for spreading COVID-19. Hot-spot analysis can complement classical epidemiological and surveillance approaches, shedding light on COVID-19 spatio-temporal trends and the possible evolution of its trajectories. Furthermore, the hot-spot analysis enables easy visualization of data in a way that is accessible for stakeholders and helps them in the decision-making process.

#### Implementation of hot-spot analysis into susceptible-infected-recovered-death (SIRD) model

A problem encountered in modeling the COVID-19 pandemic is that SIRD models generally function stochastically (random $\beta$ dependent spread through susceptible population). However, pockets of cases developing usually in high density areas undergo independent, rapid infection that does not fit into larger model. This micro-system cluster is referred to as a hot-spot and undergoes independent non-stochastic hot-spot dynamics. In order to classify a specific group of cases in an area as a hot-spot the cases must first be grouped and their characteristics modeled, using each groupings characteristics to define a hot-spot cluster.

It therefore, follows that in order to produce informative predictions for governmental policy- and decision-makers, such as estimate numbers of hospital beds, use of intensive care units (ICUs) wards and when the peak will occur, the hot-spot cluster cases must be extracted from the data the stochastic SIRD model is calibrated on. The model is then able to interpret the progression of COVID-19 without the inconsistencies incurred by the non-conforming hot-spot cases.

This is done by extracting the daily ratio of stochastic cumulative cases from the total cases in all clusters and applying this ratio to the recorded data before it is used to inform the model:

8 $$\begin{aligned} I_{stoch} = \frac{I_s}{I_s + I_{hs}} I_d, \end{aligned}$$

where $I_{stoch}$ is the stochastic active cases, $I_s$ is the active cases in stochastic clusters, $I_{hs}$ is the active cases in hot-spot clusters and $I_d$ is the active cases recorded.

### Study replication considerations

In this study, unsupervised clustering is combined with an epidemiological analysis, in order to expose the spatio-temporal virus dynamics within Gauteng Province. Although infections grouped within the same cluster do not necessarily share temporality or contact network, the methodology provides important insight into the spatio-temporal distribution of cases within the area at an improved granularity. As the methodology presented is data-driven, it can be applied to any location experiencing an epidemic, if there is sufficient data. When implementing this methodology, the number of clusters used is selected to provide the desired cluster granularity for the given area. The study can therefore be applied to any area large enough to have sufficient case data for analysis. The calibration of the hot-spot definitions must consider the generalized virus progression over the entire area as well as the socio-economic and political subtleties of the area. If the location of analysis has more comprehensive data available (such as socio-economic, movement and exposure of infected), the method can be expanded to provide more complex and/or specific insight.

## Conclusion

Hot-spot analysis represents an advanced statistical approach that can be effectively utilized for outbreak analytics and visualization. It can equip public health policy- and decision-makers with updated, real-time assessment of the pandemic trends and its future projected trajectories. Furthermore, it can complement classical epidemiological surveys, leading to the identification of patterns that would be otherwise classified as low-risk ones. In conclusion, hot-spot analysis has been highly helpful in promptly recognizing high-risk clusters, and to adopt/adjust proper public health measures. Since epidemics are situations which are highly changeable and constantly under flux, we can anticipate that hot-spot analysis can aid stakeholders in making informed, evidence-based and data-driven decisions, during epidemic waves and efforts such as vaccine roll-outs.

## Data Availability

The data that support the findings of this study are available from the Provincial Government of Gauteng (the owner of the data and a co-author in our manuscript) but restrictions apply to the availability of these data, which were used under license for the current study, and so are not publicly available. Data are however available from the authors upon reasonable request and with permission of the Provincial Government of Gauteng.
